# Quantification of T4-Like and T7-Like Cyanophages Using the Polony Method Show They Are Significant Members of the Virioplankton in the North Pacific Subtropical Gyre

**DOI:** 10.3389/fmicb.2020.01210

**Published:** 2020-06-16

**Authors:** Svetlana Goldin, Yotam Hulata, Nava Baran, Debbie Lindell

**Affiliations:** Faculty of Biology, Technion – Israel Institute of Technology, Haifa, Israel

**Keywords:** virus, cyanophage, quantification, polony, NPSG, *Prochlorococcus*, depth profile, cyanobacteria

## Abstract

The North Pacific Subtropical Gyre (NPSG) is one of the largest biomes on Earth, with the cyanobacterium *Prochlorococcus* being the most abundant primary producer year-round. Viruses that infect cyanobacteria (cyanophages) influence cyanobacterial mortality, diversity and evolution. Two major cyanophage families are the T4-like cyanomyoviruses and T7-like cyanopodoviruses, yet their abundances and distribution patterns remain unknown due to difficulty in quantifying their populations. To address this limitation, we previously adapted the polony method (for PCR colony) to quantify T7-like cyanophages and applied it to spring populations in the Red Sea. Here, we further adapted the method for the quantification of T4-like cyanophages and analyzed the abundances of T4-like and T7-like cyanophage populations in the photic zone of the NPSG in summer 2015 and spring 2016. Combined, the peak abundances of these two cyanophage families reached 2.8 × 10^6^ and 1.1 × 10^6^ cyanophages ⋅ ml^–1^ in the summer and spring, respectively. They constituted between 3 and 16% of total virus-like particles (VLPs), comprising a substantial component of the virioplankton in the NPSG. While both cyanophage families were highly abundant, the T4-like cyanophages were generally 1.3–4.4 fold more so. In summer, cyanophages had similar and reproducible distribution patterns with depth. Abundances were relatively low in the upper mixed layer and increased to form a pronounced subsurface peak at 100 m (1.9 × 10^6^ and 9.1 × 10^5^ phages ⋅ ml^–1^ for the T4-like and T7-like cyanophages, respectively), coincident with the maximum in *Prochlorococcus* populations. Less vertical structure in cyanophage abundances was apparent in the spring profile, despite a subsurface peak in *Prochlorococcus* numbers. In the summer upper mixed layer, cyanophages constituted a smaller proportion of VLPs than below it and cyanophage to cyanobacteria ratios were considerably lower (1.3–2.8) than those of VLPs to bacteria (8.1–21.2). Differences in abundances between the two families and their contribution to VLPs with depth suggest differences in cyanophage production and/or decay processes relative to other members of the virioplankton in the upper mixed layer. These findings highlight the importance of quantifying distinct populations within the virioplankton to gain accurate understanding of their distribution patterns.

## Introduction

The North Pacific Subtropical Gyre (NPSG) is one of the largest oligotrophic oceanic expanses on Earth in which a significant proportion of global primary production occurs (Karl and Church, 2014, 2017). The photoautotrophic community in the NPSG is numerically dominated by the cyanobacterium *Prochlorococcus* year-round (Campbell et al., 1997; Malmstrom et al., 2010). *Prochlorococcus* abundances are generally two orders of magnitude higher than those of *Synechococcus*, the second most abundant cyanobacterium in these waters, and eukaryotic phytoplankton (Campbell et al., 1994, 1997; Partensky et al., 1999; Malmstrom et al., 2010; Flombaum et al., 2013; van den Engh et al., 2017). Cyanobacteria are present throughout the photic zone with their depth distribution typically displaying a subsurface maximum below the upper mixed layer (Campbell et al., 1997; van den Engh et al., 2017).

Cyanophages (viruses that infect cyanobacteria) influence cyanobacterial mortality, diversity and evolution (Proctor and Fuhrman, 1990; Waterbury and Valois, 1993; Palenik et al., 2003; Lindell et al., 2004; Avrani et al., 2011; Marston et al., 2012; Ahlgren et al., 2019). Yet little is known about cyanophages in the NPSG despite the wealth of information about the cyanobacteria in this environment. Cyanophages that infect *Prochlorococcus* and *Synechococcus* are tailed double-stranded DNA viruses belonging to the order *Caudovirales* (Waterbury and Valois, 1993; Ackermann, 2003). They include a number of distinct families: the T7-like podovirus, the T4-like myovirus, the TIM5-like myovirus, and a number of siphovirus families (Fuller et al., 1998; Sullivan et al., 2005, 2009, 2010; Pope et al., 2007; Huang et al., 2012; Sabehi et al., 2012; Labrie et al., 2013). The T4-like and T7-like cyanophages are the most commonly isolated cyanophages from the oceans and are well represented in metagenomic datasets, suggesting that they are the two major families of cyanophages in the oceans (Suttle and Chan, 1993; Waterbury and Valois, 1993; Marston and Sallee, 2003; Sullivan et al., 2003; Angly et al., 2006; DeLong et al., 2006; Millard and Mann, 2006; Bench et al., 2007; Dekel-Bird et al., 2013, 2015; Labrie et al., 2013; Chow and Suttle, 2015; Huang et al., 2015; Hanson et al., 2016; Aylward et al., 2017; Luo et al., 2017). Phylogenetic analysis of cyanophage isolates show that members of these two families are genetically diverse and consist of several discrete lineages or clades, four for the T4-like myoviruses (I to IV) and two for the T7-like podoviruses (A and B) (Zhong et al., 2002; Marston and Sallee, 2003; Sullivan et al., 2008; Wang and Chen, 2008; Dekel-Bird et al., 2013; Labrie et al., 2013).

Our knowledge of cyanophage abundances and distribution patterns is particularly sparse. This is largely due to lack of methods for their quantification at appropriate taxonomic levels (Suttle, 2005; Baran et al., 2018). For example, titering measures infective phages, but only the subset of the population that infects the particular host(s) used in the assay (Suttle, 2005; Dekel-Bird et al., 2015), quantitative PCR (qPCR) affords precise quantification of a single genotype but not of multiple genotypes (Fuller et al., 1998; Baran et al., 2018), and metagenomic surveys provide a community measure relative to other components of the community (Brum and Sullivan, 2015; Roux et al., 2016). To address this limitation we recently developed the polony method (Mitra and Church, 1999) for the quantification of T7-like cyanophages and applied it to spring populations in the Red Sea (Baran et al., 2018). This is a solid-phase, single molecule PCR method that uses degenerate primers to capture the diversity of phages present in the family of interest. Here we further developed the polony method for the quantification of the T4-like cyanophages. We then applied the methods to quantify both T4-like and T7-like cyanophages over depth profiles in the NPSG in the summer of 2015 and the spring of 2016. We found that cyanophage abundances reached 1.1–2.8 × 10^6^ viruses ⋅ ml^–1^ seawater for much of the photic zone, and made up approximately 9–13% of all virus-like particles in the upper 150 m of a 1 m^2^ integrated water column. The T4 and T7-like cyanophage families displayed similar depth distribution patterns, with the T4-like cyanophages being more abundant than T7-like cyanophages, especially in the upper mixed layer in the NPSG in summer 2015.

## Materials and Methods

### The Polony Method for Quantification of T7-Like Cyanophage Populations

Quantification of T7-like cyanopodoviruses by polonies was done as described in detail in Baran et al. (2018). Briefly, up to 2.5 μl of seawater samples containing phages (the 0.2 μm filtrate) were mixed with a 10% acrylamide solution containing an acrydite modified PCR primer (Eurofins MWG Operon) in a final volume of 11.6 μl. The gel mix was then poured to polymerize on a custom-made Teflon coated microscope slide with a 25–40 μm deep oval well (Thermo Fisher Scientific), such that each phage is spatially separated in the gel. The acrydite modification covalently attaches to the gel matrix during polymerization and thereby anchors the primer and the resulting amplicon to the gel. The gels were then washed as described in Baran et al. (2018) to remove remnants of the polymerizing agents and any inhibitors in the seawater samples. Next an unmodified primer (Sigma Aldrich) and PCR reagents are diffused into the gel. The PCR reagents consist of 0.67 U ⋅ μl^–1^ Jumpstart Taq polymerase (Sigma-Aldrich), 1× Taq buffer with magnesium chloride (Sigma-Aldrich), 0.25 mM deoxyribonucleotide triphosphate mix (Hongene Biotechnology), 0.2% BSA (Sigma-Aldrich) and 0.1% Tween-20 (Sigma-Aldrich). The phages are heat lysed to release phage DNA, which is amplified to produce a PCR colony, termed a polony, in a DNA Engine twin-tower slide thermocycler (Bio-Rad). The polonies are then detected by hybridization with Cy5 or Cy3 fluorescently labeled probes (Sigma Aldrich) that are internal to the amplicon. Polonies are then visualized using the GenePix 4000B microarray scanner (Axon Instruments) and enumerated using ImageJ software (Rasband, 1997–2018).

Polony formation efficiency, or the polony-to-phage conversion efficiency, is defined as the percent of phage particles that result in polonies. This was determined previously for five different T7-like cyanophage isolates (Baran et al., 2018) as the number of phages that resulted in polonies out of the number of phages analyzed. The number of analyzed phages was determined by quantification of VLPs in the lysates by epifluorescence microscopy (Baran et al., 2018). The average of 10,000 bootstrap resamplings of the polony efficiencies for these five T7-like cyanophages was used to calculate their abundances in field samples. Bootstrap resamplings were carried out with replacement in the stats R package (Baran et al., 2018). Confidence intervals were determined from the 95% quantiles of the bootstrap analysis.

The signature gene for T7-like cyanophages is the DNA polymerase gene (*DNApol*) and is amplified with 20 μM of the 5′-acrydite-modified 534Rd primer and 15 μM of the non-modified 341Fd-15-NNN primer (Baran et al., 2018) (see Table 1 for primer and probe information). PCR cycling was initiated with a 5 min denaturation step at 94°C, followed by 50 cycles of denaturation at 94°C for 45 s, annealing at 50°C for 45 s, elongation at 72°C for 2 min, and ending with a 6 min elongation step at 72°C. This yields an amplicon of 578–584 bp.

**TABLE 1:** Primers and probes used in this study.

**Target phage**	**Target gene**	**Name and orientation**	**Sequence**	**Modification**	**References**
**Primers**					
T7-like cyanophages	DNApol	534Rd, reverse	TGNWRYTCRTCRTGNAYRAA	5′-Acrydite	Baran et al., 2018
		341Fd-15-NNN, forward	NNNCCNAAYYTNGSNCAR	none	Baran et al., 2018

T4-like cyanophages and some non-cyano T4-like phages	g20	CPS1.2, forward	ATHTTYTAYATHGAYGTNGG	5′-Acrydite	This study
		CPS8.2, reverse	ARTAYTTNCCNRYRWANGG	none	This study

**Probes**					
T7-like cyanophages, clade A	DNApol	405AF(d+3i)	TAYTGYYTIATITAYGGIGG	5′-Cy3	Baran et al., 2018
T7-like cyanophages, clade B	DNApol	405BF(d+3i) + 405BF(d+3i)_tip42	TAYGCITTYYTITAYGGIGC + TAYTGITTYYTITAYGGIGG	5′-Cy5	Baran et al., 2018

T4-like cyanophages and some non-cyano T4-like environmental sequences	g20	Probe 1: g20_cyano_env	RTCRTAYTGDATRTGITC	5′-Cy5	This study
Some non-cyano T4-like environmental sequences	g20	Probe 2: g20_env	GCRAARTRICCRTCYTK	5′-Cy3	This study

A combination of degenerate clade-specific hybridization probes was used for amplicon detection (Baran et al., 2018). Slides were hybridized with the clade B specific probes, 0.45 μM 405BF(d+3i) + 0.15 μM 405BF(d+3i)_tip42 Cy5-labeled probes, and the clade A specific probe 1.2 μM of the 405AF(d+3i) Cy3-labeled probe (Table 1). Prior to hybridization the amplicons were denatured at 70°C for 15 min in a 70% formamide solution. The hybridization mix containing the probes was then added and the gels heated to 94°C for 6 min, followed by hybridization at 42°C for 30 min. The slides were washed three times for 30 min in wash buffer E (10 mM Tris pH 7.5, 50 mM KCl, 2 mM EDTA pH 8.0, 0.01% Triton X-100). If a high level of fluorescence background was still present, an additional wash was done overnight. This wash does not change the number of polonies detected, but the reduction in background fluorescence makes it easier to count them. At least two technical replicates were carried out for each sample. Both positive and negative control slides are included in each polony experiment. The positive control contained a known concentration of the Syn5 phage diluted in 0.02 μm filtered seawater and is used to ensure that the method is working at the expected efficiency. The negative (no template) controls contained 0.02 μm filtered seawater and lacked phage or seawater samples.

### Development of the Polony Method for T4-Like Cyanophage Populations

#### Primer and Probe Design for Polony Assays

The signature gene targeted for quantification of T4-like cyanophages was the *g20* portal protein gene. In order to encompass the diversity among T4-like cyanomyoviruses, the CPS1.2 and CPS8.2 degenerate primers (Table 1) were designed based on 18 sequenced T4-like cyanophages whose genomes had been fully sequenced (Mann et al., 2005; Sullivan et al., 2005, 2010; Weigele et al., 2007; Millard et al., 2009; Doron et al., 2016). These primers were verified as suitable bioinformatically for an additional 18 T4-like cyanophage *g20* sequences (see Supplementary Table 1) as primers present in our degenerate mix match sequences that amplified them. The CPS1.2 forward and CPS8.2 reverse primers generate an amplicon of 586–595 bp in length and have degeneracies of 288 and 4096, respectively.

Degenerate hybridization probes were designed to differentiate between T4-like cyanophages and other T4-like phages and were then tested empirically. A multiple sequence alignment [ClustalX version 2.1, Larkin et al., 2007) of the *g20* gene was generated from 125 T4-like cyanophage sequences (93 cyanophage isolates and 32 environmental sequences that cluster with T4-like cyanophage isolates) and 64 non-cyano T4-like phage sequences (22 phage isolates and 42 environmental sequences). The T4-like cyanophages were isolated from a variety of habitats including marine, estuarine and fresh waters and a variety of seas and oceans, including both coastal and open ocean sites. The non-cyano T4-like phages consisted of representatives from the near T4 phages, including T-evens, Pseudo and Schizo T-evens and Exo T-evens, as well as Far T4 phages to cover the diversity of the large T4 phage group (Desplats and Krisch, 2003). Sequences for T4-like cyanophage and non-cyano T4-like environmental sequences were also collected from diverse habitats. All sequences were obtained from NCBI’s GenBank. A combination of two probes was designed: The first probe, the 5′-Cy5 labeled Probe 1:g20_cyano_env, to detect all T4-like cyanophages as well as a single clade of non-cyano T4-like environmental sequences; and a second probe, the 5′-Cy3 labeled Probe 2:g20_env, to detect this same clade of non-cyano T4-like environmental sequences. These probes are complementary to the 5′-acrydite anchored strand and have degeneracies of 48 (Probe 1:g20_cyano_env) and 64 (Probe 2:g20_env) (Table 1).

**FIGURE 1:**
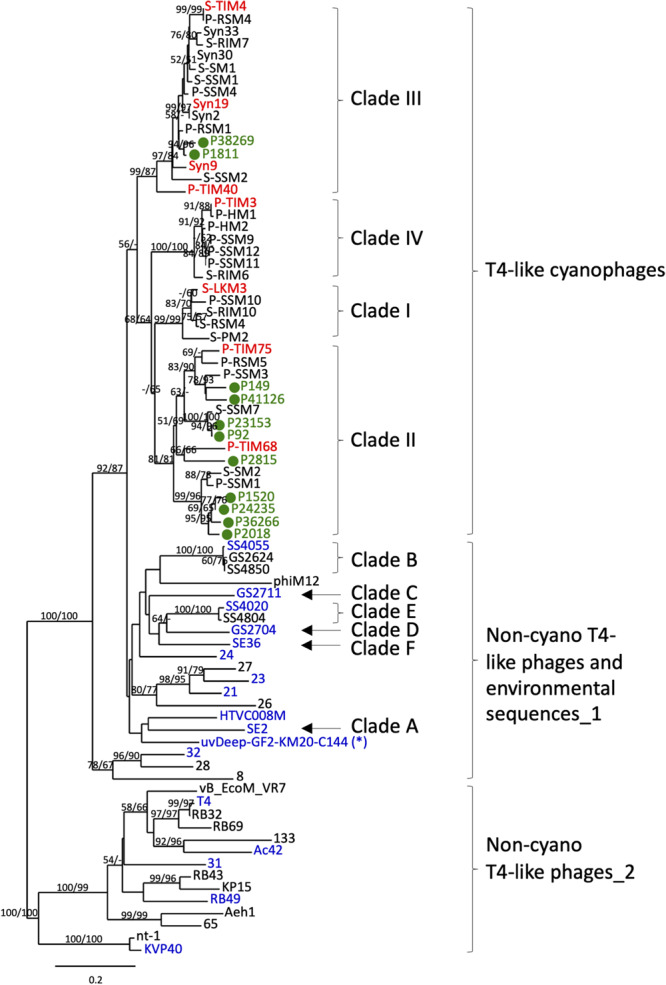
Phylogenetic identification of T4-like cyanophage polonies from environmental samples. Neighbor-joining (NJ) tree of the *g20* gene product inferred from 182 amino acid positions from T4-like cyanophage isolates, non-cyano T4-like phage isolates, non-cyano T4-like environmental sequences and sequenced polonies amplified in this study from the summer 2015 cruise. The sequenced polonies, shown in green and labeled as P#, clustered with known T4-like cyanophages. The T4-like cyanophage isolates used for empirical testing of primer and probe specificity are shown in red and non-cyano T4-like phages and environmental sequences used for empirical testing of primer and probe specificity are shown in blue (see Table 2). The single non-cyano clade detected with both Probe 1 and Probe 2 (see Table 2) is marked with *. The neighbor-joining (NJ) tree was constructed from 182 amino acid positions of T4-like phages. Bootstrap values greater than 50% for NJ and maximum likelihood (ML) trees are shown at the nodes (NJ/ML). See Supplementary Table 1 for NCBI accession numbers for all sequences in the tree. The scale bar indicates the number of amino acid substitutions per site.

The specificity of these primers and probes was empirically tested first by regular PCR and then in the polony assay with representative phages from the T4 group (Table 2). These included eight T4-like cyanophages from all four clades (shown in red in Figure 1) as well as seventeen non-cyano T4-like phages and environmental sequences selected to represent the diversity among T4-like phages (shown in blue in Figure 1). The assays were carried out with phage lysates for T4-like cyanophages: S-LKM3 (clade I), P-TIM75 and P-TIM68 (clade II), Syn9, Syn19, S-TIM4 and P-TIM40 (clade III), P-TIM3 (clade IV), and for the T4 phage itself. The other non-cyanophage T4-like phage sequences were tested after cloning into plasmids. Four non-cyano T4-like environmental sequences from Lake Kinneret, Israel, were amplified using the CPS1.1 and CPS8.1 primers and cloned in the TOPO TA plasmid (Invitrogen). For the other T4-like phages and environmental sequences a 592–625 bp fragment of the gene was synthesized (GENEWIZ) that included the region amplified by the CPS1.2 and CPS1.8 primers with 15 bp upstream and downstream of this region when the sequence was available. The synthesized gene fragments were cloned into the pUC57 plasmid (GENEWIZ) and used in PCR and polony experiments. These non-cyano T4-like phages were HTVC008M, RB49, KVP40, 31, Ac42 and the non-cyano T4-like environmental sequences were SE2, GS2704, SE36, SS4055, SS4020, GS2711, uvDeep-GF2-KM20-C144 (see Table 2 for details).

**TABLE 2:** Specificity of primers and probes for polony detection of T4-like cyanophages.

**Phage or sequence name (clade designation)**	**Site of isolation/collection**	**References**	**Amplification with *g20* primers**	**Detection with**	
				***g20* probes**	
				**Probe 1**	**Probe 2**
**T4-like cyanophages**					
S-LKM3 (Clade I)	Lake Kinneret, Station A, Israel	This study	+	+	–
P-TIM75 (Clade II)	Gulf of Aqaba, IUI pier, Red Sea	Zborowsky and Lindell, 2019	+	+	–
P-TIM68 (Clade II)	Kiribati: Caroline Island, Pacific Ocean	Fridman et al., 2017	+	+	–
Syn9 (Clade III)	Woods Hole Harbor, Atlantic Ocean	Waterbury and Valois, 1993	+	+	–
P-TIM40 (Clade III)	Line Islands, Pacific Ocean	Doron et al., 2016	+	+	–
Syn19 (Clade III)	Sargasso Sea	Waterbury and Valois, 1993	+	+	–
S-TIM4 (Clade III)	Gulf of Aqaba, IUI pier, Red Sea	Avrani et al., 2011	+	+	–
P-TIM3 (Clade IV)	Gulf of Aqaba, Station A, Red Sea	Avrani et al., 2011	+	+	–
**Non-cyano T4-like phages**					
T4	Likely from feces-containing sewage (see Abedon, 2000)	Miller et al., 2003	–	–	–
HTVC008M	Bermuda Hydrostation S	Zhao et al., 2013	–	–	–
RB49	Long Island, NY, USA	Monod et al., 1997	–	–	–
KVP40	Urado Bay, Kochi, Japan	Matsuzaki et al., 1992	–	–	–
31	Ariege, France	Petrov et al., 2006	+	–	–
Ac42	Data not available	Petrov et al., 2010	+	–	–
**Non-cyano T4-like environmental sequences**					
SE2	Savannah estuary	Zhong et al., 2002	+	–	–
GS2704	Gulf Stream	Zhong et al., 2002	+	–	–
SE36	Savannah estuary	Zhong et al., 2002	+	–	–
SS4055	Sargasso Sea	Zhong et al., 2002	+	–	–
SS4020	Sargasso Sea	Zhong et al., 2002	+	–	–
GS2711	Gulf Stream	Zhong et al., 2002	+	–	–
21	Lake Kinneret, Station A, Israel	This study	+	–	–
23	Lake Kinneret, Station A, Israel	This study	+	–	–
24	Lake Kinneret, Station A, Israel	This study	+	–	–
32	Lake Kinneret, Station A, Israel	This study	+	–	–
uvDeep-GF2-KM20-C144	Ionian Sea, Mediterranean Sea	Mizuno et al., 2016	+	+	+

#### Polony Procedures

Polony procedures for the T4-like cyanophages were similar but not identical to those described above and in Baran et al. (2018) for the T7-like cyanophages. In the polony method for the T4-like cyanophages, seawater samples were treated prior to polony assays with 50 mM EDTA for 30 min at 65°C to make the encapsidated DNA accessible to polony amplification (see section Results). Up to 2.5 μl of treated seawater samples were added to gels in a final volume of 11.6 μl. The gels were washed twice in MilliQ water and once in 0.025% Tween-20 (see Baran et al., 2018) to remove all potential PCR inhibitors including the EDTA, those in seawater and any remaining polymerization reagents. Amplification was carried out with 10 μM of the 5′-acrydite-modified CPS1.2 and 10 μM of the non-modified CPS8.2 primer. PCR cycling was initiated with a 5 min denaturation step at 94°C, followed by 50 cycles of denaturation at 94°C for 45 s, annealing at 35°C for 45 s, ramping at 0.3°C ⋅ s^–1^ to 50°C (as per Sullivan et al., 2008), elongation at 72°C for 2 min, and a final elongation step at 72°C for 6 min. Polonies from the T4-like cyanophages and one clade of non-cyano T4-like environmental sequences were detected using 0.6 μM of Probe 1:g20_cyano_env. The same clade of non-cyano T4-like environmental sequences was detected with 1.2 μM of Probe 2:g20_env (see Table 2). T4-like cyanophage polonies were counted as those detected with Probe 1 minus the polonies detected with both Probe 1 and Probe 2. Non-specific polonies detected with Probe 2 alone were not counted. At least two technical replicates were carried out for each sample. Both positive and negative control slides were included in each polony experiment. The positive control contained a known concentration of the Syn9 phage diluted in 0.02 μm filtered seawater and was used to ensure that the method is working at the expected efficiency. The negative (no template) control contained 0.02 μm filtered seawater and lacked phage or seawater samples.

The number of T4-like cyanophages in seawater samples was calculated from the average of 10,000 bootstrap resamplings using the polony formation efficiencies of six T4-like cyanophages (see Results), as done for the T7-like cyanophages (Baran et al., 2018). Bootstrap resamplings were carried out with replacement in the stats R package. Confidence intervals were determined from the 95% quantiles of the bootstrap analysis. Accurate quantification is achieved when 10–2000 polonies are present per slide. This corresponds to 10^4^ – 2 × 10^6^ T4-like cyanophages per ml of seawater. When fewer polonies are present we suggest concentrating seawater samples using a modified iron flocculation method, and when more polonies are present, the sample should be diluted in 0.02 μm filtered seawater (Baran et al., 2018).

Polony formation efficiency was tested for the T4-like cyanophages with and without treatment in 50 mM EDTA for 30 min at 65°C. Six phages belonging to the four different T4-like cyanophage clades were used: S-LKM3, P-TIM75, P-TIM68, Syn9, P-TIM40 and P-TIM3 (see Figure 1 for clade designations). The number of virus-like particles in each lysate was determined by epifluorescence microscopy (see below).

#### Regression Curve Analysis

Regression curve analysis of phage-to-polony conversion was carried out for the Syn9 phage after the EDTA-heat treatment (as described above). The number of input phages was determined by virus-like particle counts using epifluorescence microscopy as described below. Major axis regression was carried out by using the Lmodel2 package in R (R Core team, 2013). No polonies were observed for the no template control samples, therefore, intercepts were set to zero.

#### Polony Picking and Sequencing

Eleven polonies were picked from the summer depth profile collected on 30 July 2015. Two depths were chosen; 25 m in the upper mixed layer where the abundance of T4-like cyanophages was relatively low, and 75 m where the peak in T4-like cyanophage abundances was observed. The picking procedure for the T4-like polonies was similar to that of the T7-like polonies (Baran et al., 2018), but with primers and PCR conditions for the T4-like cyanophages. A picked piece of gel containing a polony was placed in a 39 μl PCR reaction volume containing 1× Taq PCR MasterMix (TIANGEN) and 1.25 μM of both CPS1.1 and CPS8.1 primers. We employed the CPS1.1/CPS8.1 primer set for the picking procedures since polony amplification efficiency was higher for these less degenerate primers than for the CPS1.2 and CPS8.2 primers used for the polony assays. Thermocycling consisted of an initial denaturation step of 5 min at 94°C, 36 cycles of denaturation at 94°C for 30 s, annealing at 35°C for 1 min, ramping at 0.3°C ⋅ s^–1^ to 50°C, elongation at 72°C for 1 min and a final elongation step at 72°C for 5 min. The resulting PCR amplicons were either extracted from the agarose gel or cleaned directly from the reaction mixture using PCR clean-up and gel extraction kit (MACHEREY-NAGEL). Next, these *g20* amplicons were cloned using the PCRII Topo TA cloning kit (Invitrogen) and transformed by electroporation into competent DH10B *E. coli* cells. Plasmid inserts were then sequenced by Sanger sequencing. The polony sequences were submitted to NCBI and have the following accession numbers: MN701566-MN701576.

#### Phylogenetic Analysis

Multiple sequence alignment of translated *g20* genes (182 amino acid positions) was done with ClustalX version 2.1 (Larkin et al., 2007) and visually verified. Neighbor-joining (ProtDist/FastDist and BioNJ) and maximum likelihood (PhyML) phylogenetic trees were generated using the Phylogeny.fr website (Dereeper et al., 2008). Bootstrap analyses were carried out with 1000 resamplings for the neighbor-joining tree and 500 for the maximum likelihood tree.

### Virus-Like Particle (VLP) Quantification

Virus-like particle enumeration was done as described by Patel et al. (2007). In brief, 1–4 ml of lysate or seawater were filtered onto an Anodisc 25 mm 0.02 μm pore-sized filter (Whatman) and stained with SYBR Green I at a dilution of 1:100. The filter was then visualized using epifluorescence microscopy (Zeiss Axiovert 200 inverted microscope) with the GFP filter set (Ex: 470/40 nm; Em: 525/50 nm) at 1000x magnification. VLPs were counted automatically using the openCFU (3.9.0) software (Geissmann, 2013). Two fields from each slide were also counted manually to verify suitable settings for automatic counting. For analysis of VLP abundances in the NPSG and their comparison to cyanophage abundances determined by the polony method, we assumed that the vast majority of VLPs were made up of dsDNA containing viruses and not extracellular vesicles (Biller et al., 2017).

### Study Site and Sample Collection

**FIGURE 2:**
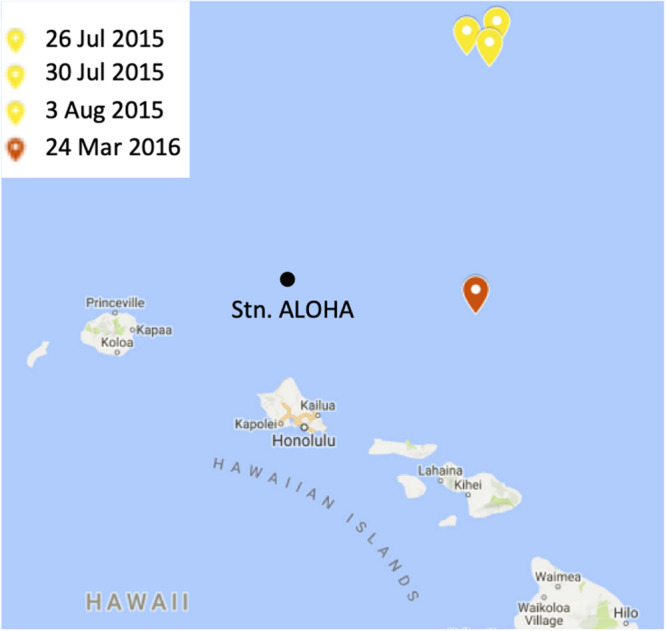
A map of the study site. The locations and dates of sampling during the summer 2015 and spring 2016 cruises are indicated in yellow and red, respectively. Station ALOHA is shown for context. The figure was made in Microsoft PowerPoint 2013 by modifying Hawaiian Islands Google Map (Map data ©2019, Google). https://www.google.com/maps.

Seawater samples were collected from two cruises conducted in the North Pacific Subtropical Gyre (Figure 2). HOE-Legacy (HL) 2 and 3 were held between 24 July to 5 August 2015 and 23–28 March 2016, respectively. Both cruises were on the *R/V Kilo Moana* and employed a Lagrangian sampling strategy in anti-cyclonic eddies (Wilson et al., 2017). Sampling was conducted using a 24 X 12 L Niskin bottle rosette attached to a conductivity-temperature-depth (CTD) package (SBE 911 plus, SeaBird) and fitted with fluorescence, oxygen and transmissometer sensors (Wilson et al., 2017). Samples were collected from three profiles over a 12-day period during the summer 2015 cruise and from a single profile during the spring 2016 cruise.

Samples for the enumeration of the cyanobacteria, *Prochlorococcus* and *Synechococcus*, and heterotrophic bacteria by flow-cytometry were collected and analyzed as per the Hawaii Ocean Time Series program (Wilson et al., 2017). Briefly, the cyanobacteria were counted based on size and the autofluorescence of chlorophyll *a* and phycoerythrin for *Prochlorococcus* and *Synechococccus*, respectively. Total bacteria were enumerated after staining with the SYBR Green I DNA stain and heterotrophic bacteria were determined to be the difference between total bacteria and the cyanobacteria. All data are available at http://hahana.soest.hawaii.edu/hoelegacy/data/data.html.

Water samples for VLPs and polony analyses were collected from eight discrete depths in the photic zone (5, 15, 25, 45, 75, 100, 125, 150 m). Water was pre-filtered over a 20 μm mesh. Samples were then filtered through a 0.2 μm PVDF Millex-GV syringe filter (Merck Millipore) and the filtrate was collected. Filtrate samples for cyanophage quantification using the polony method were frozen at −80°C with no fixative. Samples for VLP quantification were fixed in 0.7% formaldehyde (Bio-Lab) prior to freezing at −80°C.

### Statistics

*T*-tests were used to determine the significance of EDTA-heat treatment on T4-like cyanophage lysates as well as on T4-like and T7-like cyanophages in seawater samples from a number of environments. Shapiro–Wilk test was employed to test whether the data were normally distributed. When normally distributed (S-LKM3, P-TIM75, P-TIM68, Syn9, P-TIM40 cyanophages) two-sample two-tailed *t*-tests were carried out. When not normally distributed (P-TIM3), the Mann–Whitney *U*-Test was used. The data for the environmental seawater samples were normally distributed and two-tailed, paired *t*-tests were used to compare untreated and EDTA-heat treated samples.

## Results

### Development of the Polony Method for Quantification of T4-Like Cyanophages

The polony method is based on solid-phase PCR amplification of phage DNA from individual virions. The virions collected from the virus fraction (<0.2 μm) of seawater samples are embedded in an acrylamide gel together with an acrydite modified primer that serves to anchor the resultant PCR amplicons to the gel (Baran et al., 2018). These amplicons (PCR colonies or polonies) are then detected by hybridization with fluorescently labeled probes internal to the primers (see section “Materials and Methods”).

#### Specificity of Primers and Probes for Polony Quantification of T4-Like Cyanophages

Quantification of phages at the family level requires that the combination of primers and probes used be specific for the phage family of interest, yet cover the diversity found within the family. For the T4-like cyanophages we targeted the portal protein gene, *g20*, since this virion structural gene is found in all T4-like cyanophages and is considered a signature gene for T4-like phages (Sullivan et al., 2008). Furthermore, the sequence diversity of this gene is well-known both for the T4-like cyanophages and for the larger T4-like group (Fuller et al., 1998; Zhong et al., 2002; Marston and Sallee, 2003; Sullivan et al., 2008; Marston and Amrich, 2009), enabling appropriate design of primers and probes for the amplification and detection specifically of T4-like cyanophages. We designed primers based on 18 genome-sequenced T4-like cyanophage isolates and verified their suitability for other T4-like cyanophages (see section “Materials and Methods,” Supplementary Table 1). The best primers were at the same positions as the CPS1/CPS1.1 and CPS8/CPS8.1 primers used in the above mentioned diversity studies. Our primers, CPS1.2 and CPS8.2, are shorter and more degenerate and, based on bioinformatic analyses, better capture the diversity of T4-like cyanophage family as known currently. We empirically verified that these primers amplify the *g20* gene from eight T4-like cyanophages belonging to all 4 cyanophage clades (Figure 1 and Table 2). In addition, these and other previously used primer sets (CPS1/8, CPS1.1/8.1 and CPS1.2/8.2), amplify some non-cyano T4-like phages and environmental sequences (Table 2) (Zhong et al., 2002; Sullivan et al., 2008; Gao et al., 2016). Therefore, it was necessary to use probes to differentiate between T4-like cyanophages and these other T4-like phage sequences. Probe design was based on 125 T4-like cyanophage isolates and uncultivated T4-like cyanophage sequences from diverse environments that fall within the same phylogenetic clades as the isolates and 65 non-cyano T4-like phage and environmental sequences (see section “Materials and Methods”). A combination of probes was used to empirically test the specific detection and quantification of T4-like cyanophages (Table 2). Probe 1 detected all T4-like cyanophages and one clade of non-cyano T4-like environmental sequences (Figure 1 and Table 2). Probe 2 detected only this one clade of non-cyano T4-like environmental sequences (Figure 1 and Table 2). Therefore, specific quantification of T4-like cyanophages was achieved from the total number of polonies detected with Probe 1, less the number of polonies detected with both Probe 1 and Probe 2. In the NPSG samples analyzed in this study, 90–99% of the T4-like polonies per sample were cyanophages (i.e., were detected with Probe 1 but not with Probe 2).

In order to further verify that polonies produced from field samples resulted from T4-like cyanophages, we picked and sequenced 11 polonies from samples collected from the summer 2015 cruise in the NPSG. All sequenced polonies clustered with known T4-like cyanophage sequences in a phylogenetic tree of *g20* sequences (Figure 1). In particular, they clustered with clade II and III T4-like cyanophages. While this small number of sequenced polonies cannot be used to make a quantitative assessment of the composition of the different cyanophage clades detected with the polony method in these waters, it is interesting to note that Huang et al. (2015) found that metagenomic reads from T4-like cyanophages belonging to clades II and III were more common throughout the oceans than from clades I and IV. The combined primer, probe and polony sequencing results demonstrate that the polony method developed here specifically detects T4-like cyanophages.

#### Optimization of Polony Procedures for T4-Like Cyanophages

**FIGURE 3:**
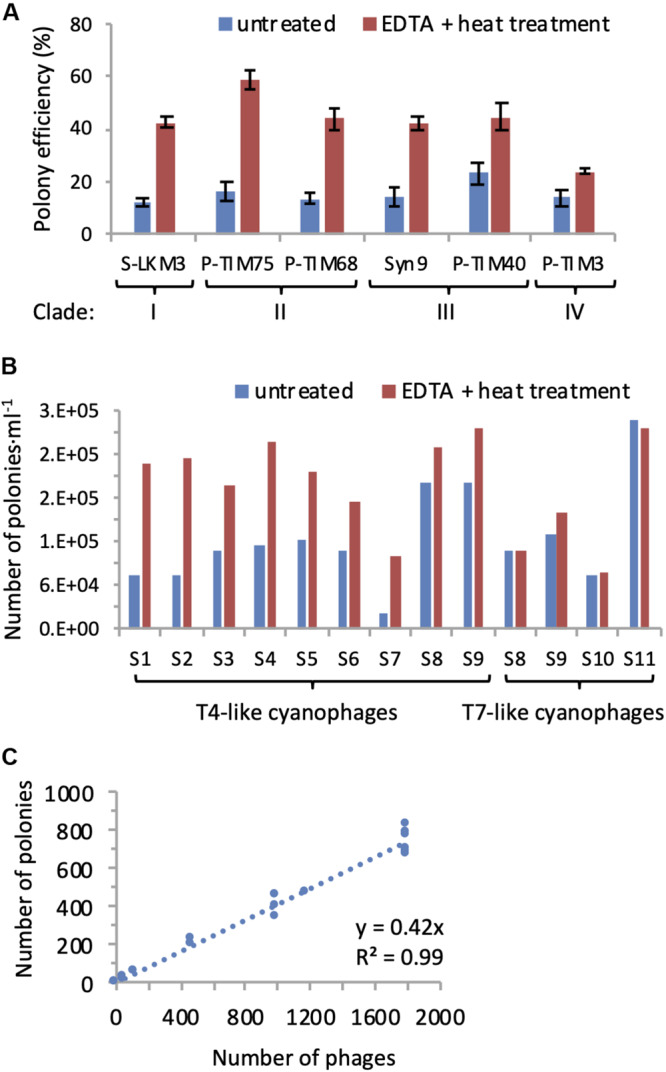
EDTA-heat treatment increases efficiency of polony formation for T4-like cyanophages. **(A)** Polony formation efficiencies determined for cyanophages from all four clades of T4-like cyanophages with and without EDTA-heat treatment. Averages and standard deviations of 5 replicate treatments are shown for each cyanophage. A significant improvement in polony formation efficiency resulted from EDTA-heat treatment for each T4-like cyanophage (two-sample two-tailed *t*-test: *p* = 3.5E-11 for S-LKM3, *p* = 2.5E-08 for P-TIM75, *p* = 2.4E-08 for P-TIM68, *p* = 2.0E-07 for Syn9, *p* = 8.1E-06 for P-TIM40; non-parametric Mann–Whitney *U* Test: *p* = 0.00804 for P-TIM3). **(B)** The number of T4-like and T7-like cyanophages detected by polonies with and without EDTA-heat treatment for samples collected from the Red Sea (samples 1–6), Mediterranean Sea (sample 7), and the North Pacific Ocean (samples 8–11). Averages of two replicates assays are shown for each sample. EDTA-heat treatment significantly increased the number of T4-like cyanophages detected by the polony assay (*p* = 9.4E-05, two-tailed paired *t*-test), but had no effect on the number of T7-like cyanophages detected (*p* = 0.519, two-tailed *t*-test). **(C)** Standard curve of polony formation for the T4-like cyanophage, Syn9, after EDTA-heat treatment (*n* = 19).

Polony conditions were optimized to obtain increased polony formation efficiency (the percent phage particles that resulted in polonies) and detection. This was done for primer and probe concentrations and was tested on six phages belonging to the four different T4-like cyanophage clades. In addition, ramping PCR (see section “Materials and Methods”) improved polony formation efficiency approximately 9-fold for phages from clade II. Despite these optimizations, efficiencies remained between 12 and 23% (Figure 3A). This is significantly lower than those reached after primer and probe optimization for the T7-like cyanophages (Baran et al., 2018). Therefore, we tested polony efficiency for naked DNA from the T4-like cyanophage, Syn9, and found that it was significantly higher than that for intact Syn9 phage particles (data not shown). These results suggested that encapsidated T4-like cyanophage DNA was not fully accessible for PCR amplification.

To overcome the above problem, we tested two approaches for making encapsidated DNA from the T4-like cyanophages more accessible to PCR. Multiple freeze-thaw cycles were tried but did not improve polony formation efficiency (data not shown). Previous studies have shown that heat treatment in the presence of EDTA destabilizes the capsids of a number of bacterial and eukaryotic viruses and leads to genome release at temperatures well below those for capsid denaturation (Duda et al., 2009; White et al., 2012; Bauer et al., 2015). Therefore, we implemented a combined EDTA and heat treatment (see section “Materials and Methods”) with our six T4-like cyanophages prior to the polony assay to assess the impact on polony formation. This treatment significantly increased polony formation efficiency for all phages tested (*p* < 0.01), ranging between a 1.7 to 3.6-fold improvement (Figure 3A). The application of the EDTA-heat treatment to seawater samples from a number of environments showed it to be very effective for the T4-like cyanophages (*p* = 9.4E-05, *n* = 9), yet unnecessary for the T7-like cyanophages (*p* = 0.52, *n* = 4) (Figure 3B). Thus, we used it for the quantification of T4-like cyanophage in all subsequent polony assays.

Next, we assessed the reproducibility and linear range of the method. A strong linear relationship between the number of polonies produced and the number of Syn9 phages was found over two orders of magnitude of input phage (Figure 3C). In addition, polony formation efficiencies were highly reproducible for each of the six T4-like cyanophages (*n* = 5 for each phage) (Figure 3A). These results demonstrate the high reproducibility and accuracy of the polony method for the T4-like cyanophages.

At the end of these optimizations, the virus-to-polony formation efficiencies for the 6 T4-like cyanophages belonging to the four different clades ranged from 24 to 59%, with 4 of them having efficiencies of approximately 40% (Figure 3A). Since we do not know the composition of the community and the contribution of different types of T4-like cyanophages, we used the average of 10,000 bootstrap resamplings of these polony efficiencies, with replacement, to calculate the abundances of the T4-like cyanophages in field samples, as per Baran et al. (2018). The potential abundances for any population could range up to twofold. If the populations were made up mainly of T4-like cyanophages with the highest polony formation efficiency, the values would be at the lower end of this twofold range, while if the composition was made up mainly of phages with the least efficient polony formation efficiency, then field abundances would be at the higher end of this twofold range. Since most T4-like cyanophages have a similar intermediate polony formation efficiency (Figure 3A), these two outer limits are unlikely to represent the populations in nature but are rather likely to be of intermediary abundances as found using the bootstrapping approach.

### Water Column Conditions and Cyanobacterial Depth Distribution in the NPSG

Next we used the polony method to investigate T4-like and T7-like cyanophage abundances in the upper 150 m of the oligotrophic waters of the NPSG. This was done for three depth profiles collected over a 12-day period in the summer of 2015 and for a single profile in the spring of 2016 (Figure 2). To place our findings in context we first describe the water column hydrography during these two cruises, as well as the depth distribution patterns of the cyanobacteria. We further compare cyanophage distribution patterns to those of viruses in general, as measured from abundances of nucleic acid stained virus-like particles (VLPs) (see section “Materials and Methods”).

**FIGURE 4:**
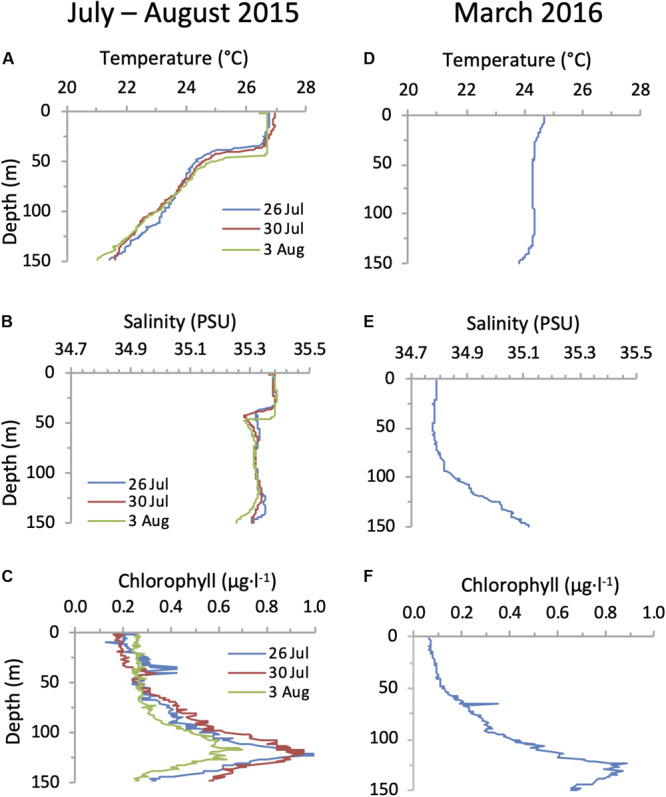
Water column conditions in the NPSG. Depth profiles of temperature **(A,D)**, salinity **(B,E)** and chlorophyll **(C,F)** during the summer cruise in July–August 2015 (left panels) and in the spring cruise on the 24 March 2016 (right panels).

Hydrographic conditions of the water column were consistent with previous observations in the NPSG (Karl, 1999; Karl and Church, 2017). In summer (26 July to 3 August 2015) the surface mixed layer extended to a depth of 40 m and was present above a stably stratified water column, while in spring (24 March 2016) the water column was weakly stratified, with a shallow upper mixed layer (Figure 4) after what appears to have been a recent mixing event beyond 100 m depth. A deep chlorophyll maximum (DCM) of similar magnitude was observed on both cruises at a depth of approximately 125 m (Figure 4).

**FIGURE 5:**
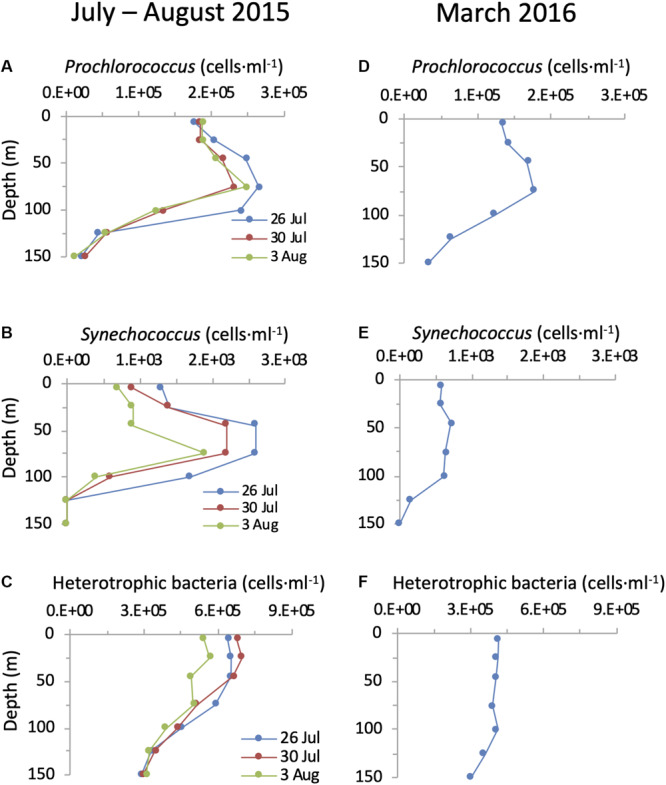
Depth distribution of cyanobacteria and heterotrophic bacteria in the NPSG. Abundances of *Prochlorococcus* **(A,D)**, *Synechococcus* **(B,E)** and heterotrophic bacteria **(C,F)** during the summer cruise in July–August 2015 (left panels) and in the spring cruise in March 2016 (right panels).

*Prochlorococcus* was approximately two orders of magnitude more abundant than *Synechococcus* throughout the photic zone and abundances of both picocyanobacterial genera were higher during the summer cruise than during the spring cruise (Figure 5). Both cyanobacterial genera displayed subsurface maxima that were above the DCM, between 45 and 100 m. The cyanobacterial maxima were less pronounced in the spring than in the summer. The highest abundances at these subsurface maxima for *Prochlorococcus* were 2.7 × 10^5^ cells ⋅ ml^–1^ and 1.8 × 10^5^ cells ⋅ ml^–1^ in the summer and spring respectively, while for *Synechococcus* they were 2.6 × 10^3^ cells ⋅ ml^–1^ and 0.7 × 10^3^ cells ⋅ ml^–1^. These abundances and depth distribution patterns are similar to those known for this region (Campbell et al., 1997; Malmstrom et al., 2010). The heterotrophic bacteria displayed a different depth distribution pattern to that of the cyanobacteria. No subsurface peak was observed on either cruise, with highest abundances in the upper mixed layer that declined below it (Figure 5). Combined, *Prochlorococcus* and *Synechococcus* made up between 21 and 35% of the total bacterial community in the upper 100 m of the water column (Figure 5).

### Depth Distribution of the T4-Like and T7-Like Cyanophage Families in the NPSG

**FIGURE 6:**
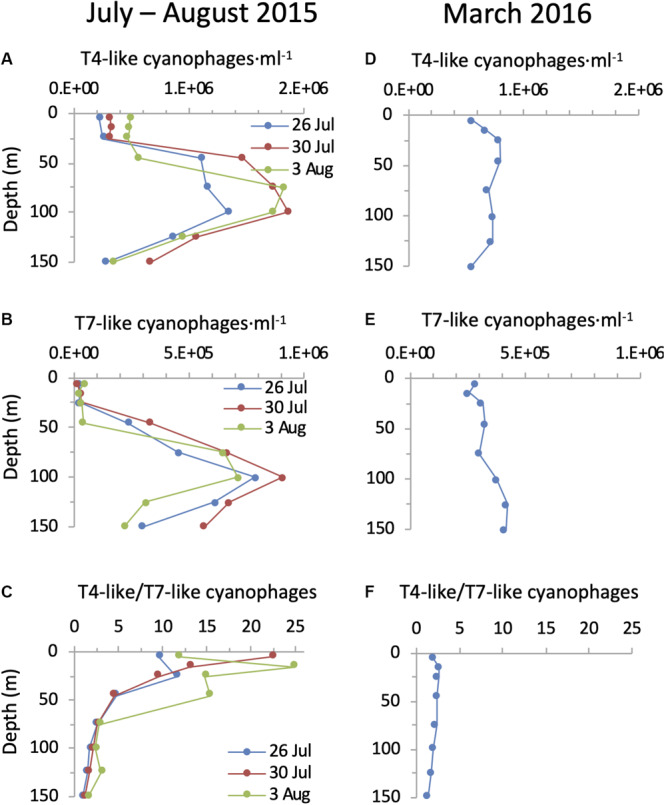
Depth distribution of cyanophages in the NPSG. Abundances of T4-like cyanophages **(A,D)**, T7-like cyanophages **(B,E)** and the ratio of T4-like to T7-like cyanophages **(C,F)** during the summer cruise in July–August 2015 (left panels) and in the spring cruise in March 2016 (right panels). T4-like and T7-like cyanophages were quantified using the polony method. Cyanophage abundances with 95% confidence intervals are presented for each profile in Supplementary Figure 1.

Using the polony method we found that both cyanophage families were highly abundant in the photic zone of the NPSG. During the summer cruise cyanophages displayed a clear and reproducible depth distribution pattern that was similar for both the T4-like and T7-like cyanophages in all three profiles (Figure 6). Abundances were low and relatively even in the upper mixed layer (T4-likes: 2.2–4.9 × 10^5^ ml^–1^ and T7-likes: 1.4–4.1 × 10^4^ ml^–1^), increasing dramatically below it to form a prominent subsurface peak. For the T4-like cyanophages this subsurface peak was broad extending from immediately below the upper mixed layer at 45 m down to 100 m and reaching a maximum of 1.3–1.9 × 10^6^ phage ⋅ ml^–1^. For the T7-like cyanophages the increase was more gradual and reached a sharper peak at 75–100 m of 7.1–9.1 × 10^5^ ml^–1^. Clade B T7-like cyanophages made up 84–100% of all T7-like cyanophages at all depths. Peaks of both cyanophages coincided with the maximum in cyanobacterial abundances and were above the DCM. Overall, the combined abundances of the T4-like and T7-like cyanophages reached a maximum of 2.1–2.8 × 10^6^ cyanophages ⋅ ml^–1^ at 75–100 m in the summer cruise. The differences in abundances between the upper mixed layer and the layers below it in summer were significant for both cyanophage families, as seen from non-overlapping 95% confidence intervals (Supplementary Figure 1). In the spring profile, depth distribution patterns of the two cyanophage families displayed only a small and non-significant increase in abundances below the surface, from 5.5 to 7.9 × 10^5^ ml^–1^ and 2.6 to 3.1 × 10^5^ ml^–1^ for the T4-like and T7-like cyanophages, respectively (Figure 6 and Supplementary Figure 1). Combined abundances of these two cyanophage families reached a maximum of 1.1 × 10^6^ cyanophages ⋅ ml^–1^ at 25–45 m in the spring cruise.

**FIGURE 7:**
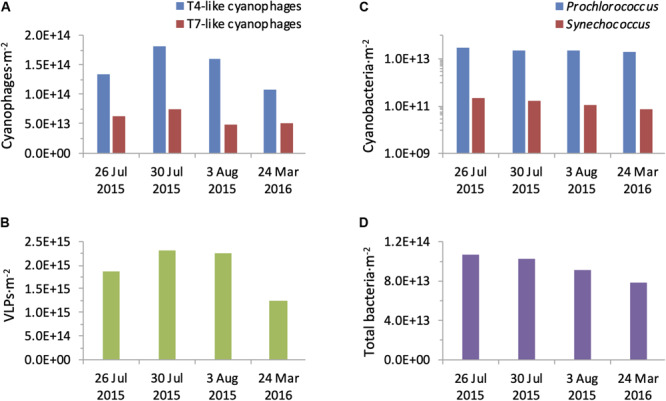
Integrated water column abundances of bacteria and viruses. Abundances of T4-like and T7-like cyanophages **(A)**, VLPs **(B)**, *Prochlorococcus* and *Synechococcus* **(C)**, and total bacteria **(D)** were calculated for a 1 m^2^ water column of 150 m depth from each profile collected during the summer 2015 and spring 2016 cruises.

The T4-like cyanophages were more abundant than the T7-like cyanophages throughout the photic zone in both summer 2015 and spring 2016 (Figure 6). This was most pronounced in the upper mixed layer in summer where the T4-like cyanophages averaged approximately 13-fold more than the T7-like cyanophages. Below the mixed layer in summer and throughout the water column in spring the difference was more subtle and quite constant, with the T4-like cyanophages being 1.6–2.8 fold more abundant than the T7-like cyanophages down to 125 m (Figure 6). Over a 1 m^2^ integrated water column of 150 m depth, T4-like cyanophages were more abundant than T7-like cyanophages by 2.1–3.2 fold (Figure 7). The overall integrated abundances in the water column were quite similar on both cruises, ranging from 1.1 to 1.8 × 10^14^ ⋅ m^–2^ for the T4-like cyanophages and 4.9 to 7.5 × 10^13^ ⋅ m^–2^ for the T7-like cyanophages.

**FIGURE 8:**
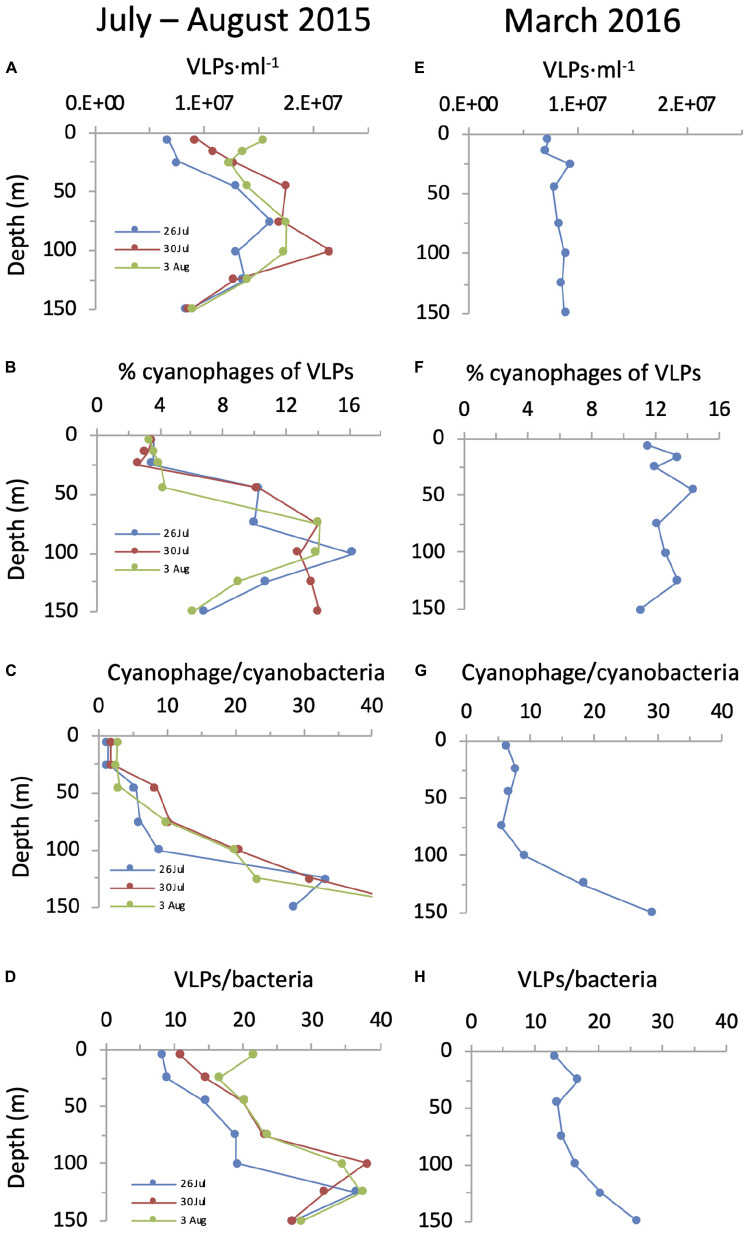
Cyanophage abundances in relation to VLPs and cyanobacteria over depth profiles in the NPSG. Abundances of VLPs **(A,E)**, percent cyanophages of VLPs **(B,F)**, the ratio of cyanophages to cyanobacteria **(C,G)** and the ratio of VLPs to total bacteria **(D,H)** during the summer cruise in July–August 2015 (left panels) and in the spring cruise in March 2016 (right panels). For these analyses, we are assuming that the vast majority of VLPs are dsDNA-containing viruses and that the majority of cyanophages in the NPSG are T4-like and T7-like cyanophages quantified by the polony method.

Next we compared cyanophage abundances to the virus community in general. For this we first enumerated virus-like-particles that detect dsDNA-containing particles in the viral fraction (Patel et al., 2007) but do not discriminate between phage families nor indicate who they infect. In summer, surface abundances were 0.6–1.5 × 10^7^ VLPs ⋅ ml^–1^ and numbers increased somewhat with depth to reach subsurface maxima of 1.6–2.2 × 10^7^ VLPs ⋅ ml^–1^ between 45–100 m (Figure 8). In spring their depth distribution was fairly constant throughout the water column with 0.7–0.9 × 10^7^ VLPs ⋅ ml^–1^. Integrated water column abundances ranged between 1.2 to 2.3 × 10^15^ VLPs ⋅ m^–2^ (Figure 7). Combined, abundances of T4-like and T7-like cyanophages made up between 3 and 16% of the VLPs throughout the water column (Figure 8), with the contribution being lowest in the upper mixed layer during the summer, such that these two cyanophage families made up between 9 and 13% of the VLPs in integrated water columns in both seasons (Figure 7).

Knowing the abundances of cyanophages enables a comparison to those of their potential cyanobacterial hosts. During the spring cruise, the ratio of T4-like + T7-like cyanophages to cyanobacteria was relatively constant from the surface down to 100 m, ranging from 5.6 to 9.0 (Figure 8). In contrast, in summer this ratio was low at the surface (1.4–2.8) and increased with depth to 8.8–20.6 at 100 m. While the ratio of VLPs to total bacteria also increased with depth (Figure 8), the ratio of cyanophage to cyanobacteria was considerably lower than that for VLPs to bacteria on both cruises, especially in the surface layers (8.1–21.2). This is despite reaching similar ratios at greater depths (Figure 8). The overall ratio within an integrated water column of cyanophages to cyanobacteria (7–10.8) were lower than those for VLPs to total bacteria (15.8–24.9).

## Discussion

Despite the NPSG being one of the largest biomes on Earth, surprisingly little is known about the abundances of viruses in this environment. A handful of previous studies have reported VLP numbers similar to those found here (Culley and Welschmeyer, 2002; Brum, 2005; Yang et al., 2010; Gainer et al., 2017), yet no information regarding cyanophage abundances or distribution patterns were known previously. The use of the polony method for the two major cyanophage families, the T4-like and the T7-like cyanophages, has shown that together they reach abundances as high as 2.8 × 10^6^ cyanophages ⋅ ml^–1^ seawater and as much as 2.57 × 10^14^ over a 1 m^2^ integrated water column in the NPSG. They make up between 9 and 13% of the general virus pool represented by VLP abundances and are thus major components of the virus community.

It is interesting to note that the relative contribution of the T4-like and T7-like cyanophages to the VLP pool (9–13%) is 2–3 fold lower than the contribution of the cyanobacteria to total bacteria (24–26.5%) over the 1 m^2^ integrated water column down to 150 m. Based on our current knowledge of cyanophage diversity, metagenomics studies indicate that the T4-like and T7-like cyanophage families are the dominant cyanophage types in the NPSG (DeLong et al., 2006; Labrie et al., 2013; Huang et al., 2015; Aylward et al., 2017), including during this same summer cruise (Aylward et al., 2017). We cannot, however, rule out the possibility that a presently unknown cyanophage family exists and is abundant in this and other oceanic regions, which would increase the relative contribution of cyanophages to the VLP pool.

The dramatic difference in the abundances of the cyanophages in the upper mixed layer relative to the layers below it in summer 2015 is intriguing, especially since the difference in cyanobacterial abundances is not nearly as large. In addition, the increase in VLPs is not as dramatic as that for the T4-like and T7-like cyanophages. Furthermore, the ratio of cyanophage to cyanobacteria is much lower in the upper mixed layer than that of VLPs to bacteria, even though they both increase with depth and reach similar values at 100–125 m (compare Figure 8C and Figure 8D). Assuming that some presently unknown cyanophage family is not highly abundant in the NPSG (see above), these observations suggest that the magnitude of the processes leading to virus standing stocks, their production (infection) and/or loss (decay), differ between the cyanophages and the average virus type in either the upper mixed layer or below it or both. This could be due to fewer cyanophages being produced per cyanobacterial cell in the upper mixed layer relative to the average virus type per bacterium, or to higher rates of decay after this production. The latter could be the case if cyanophages are more sensitive to mixing and UV light in surface layers than VLPs. The former could be the case if the higher light exposure or lower nutrient concentrations in the upper mixed layer affects cyanophage progeny production more than VLP production, perhaps through an indirect effect on the physiological state of cyanobacterial hosts (Chow and Suttle, 2015). Determining the causes for the observed differences will require experimental investigation of both production and decay of cyanophages relative to VLPs.

Clear differences in depth distribution patterns were apparent between the T4-like and T7-like cyanophages even though similar overall patterns of a subsurface peak were observed. First, T4-like cyanophages were more abundant than T7-like cyanophages throughout the photic zone in both spring and summer depth profiles. Second, the relative difference between T4-like and T7-like cyanophage abundances was much greater in the summer upper mixed layer due to considerably lower abundances of the T7-like cyanophage abundances. As such, the abundance of T7-like cyanophages increased 17–35 fold between the upper mixed layer and the subsurface peak, whereas the T4-like cyanophages increased 4–6 fold. Third, the T4-like cyanophages had a broad subsurface peak (45–100 m), matching the breadth of the *Prochlorococcus* maximum, whereas the T7-like cyanophage subsurface peak was narrower and at the deeper end of the *Prochlorococcus* maximum (75–100 m). These differences indicate that the two families respond somewhat differently to their environment.

The reasons behind the differences in abundances of the T4-like and T7-like cyanophages remain unknown and we can only speculate on the potential causes at this time. Perhaps T4-like cyanophages are more abundant because they consist of narrow and broad host-range viruses, while T7-like cyanophages have a very narrow host range (Waterbury and Valois, 1993; Sullivan et al., 2003; Dekel-Bird et al., 2015; Zborowsky and Lindell, 2019). Narrow host-range cyanophages can be expected to specialize on abundant, dominant host types whereas broad host-range cyanophages would be able to infect a range of diverse, less abundant host types (Guyader and Burch, 2008; Koskella and Meaden, 2013; Zborowsky and Lindell, 2019). As such a family of viruses with a spectrum of host-ranges, from narrow to broad, may be more abundant as they are able to infect a more diverse set of hosts than a virus family consisting of only narrow host-range specialists. In addition, the T4-like cyanophages have larger genomes and carry a larger number and a wider range of (cyano)bacterial-like genes (auxiliary metabolic genes) than the T7-like cyanophages (Mann et al., 2005; Sullivan et al., 2005, 2010; Millard et al., 2009; Labrie et al., 2013). On the one hand, a larger genome is expected to result in a smaller burst size for the same level of resources in a cell. However, if these bacterial-like cyanophage genes supplement host resources significantly more than the limited number of genes in T7-like cyanophages do, for example through enhanced nutrient uptake and/or photosynthetic energy production (Lindell et al., 2004; Zeng and Chisholm, 2012; Puxty et al., 2016, 2018), then this may nonetheless result in higher virus yields in the harsh environmental conditions present in the photic zone of the oligotrophic NPSG. The differences may also be related to the distinct sets of core replication and structural genes (Sullivan et al., 2005, 2010; Labrie et al., 2013), resulting in different life history traits, replication strategies and virion particle structures in the two families that may lead to somewhat more infection, more progeny production or longer virion stability for T4-like cyanophages under the environmental conditions in this biome. As stated above, these are all speculations at this point in time, and investigation into the physiological and genomic underpinnings of these ecological differences awaits further investigation.

The 3 depth profiles with similar abundances and depth distributions within a 12-day period in the summer, indicate that cyanophage and VLP abundances are quite stable, at least within a short period of time, and that a single profile accurately represents their distribution in the water column. Here we report results for a single summer and spring, providing first glimpses into seasonal differences in cyanophage depth distribution patterns in the NPSG. Future investigations over multiple annual cycles are needed to gain in depth understanding of their seasonal and interannual variability in this important biome.

It is difficult to compare our results to other findings of cyanophage abundances, because of both methodological and geographic differences in the datasets. Nonetheless, support for our findings of significantly higher abundances of T4-like cyanophages than T7-like cyanophages in surface waters of the NPSG in summer 2015 are found from relative values based on viromes during the same cruise (Aylward et al., 2017). In addition, abundances of T7-like cyanophages reported here for the NPSG are of a similar order of magnitude to most samples from a spring profile in the Gulf of Aqaba, Red Sea, with 1.8–7.7 × 10^5^ T7-like cyanophages ⋅ ml^–1^ that were also determined using the polony method (Baran et al., 2018). This is except for surface abundances in the NPSG in summer, which were 8 to 13-fold lower than those in the Red Sea and may be related to processes occurring in the upper mixed surface layer in the NPSG (see above).

Many previous measurements of cyanophages are culture-based, measuring infective titers on a variety of cyanobacteria. These were primarily done in other oceanic regions and focused on sites where *Synechococcus* was the dominant cyanobacterium and used *Synechococcus* strains as the assay hosts. Only a couple of studies have used *Prochlorococcus* hosts in such assays (Sullivan et al., 2003; Dekel-Bird et al., 2015). Cyanophage abundances generally ranged from hundreds to tens of thousands of cyanophages ⋅ ml^–1^ seawater in these studies (Waterbury and Valois, 1993; Suttle and Chan, 1994; Marston and Sallee, 2003; Sullivan et al., 2003; Millard and Mann, 2006; Dekel-Bird et al., 2015). Occasionally, abundances on the order of hundreds of thousands ⋅ ml^–1^ were found but these were rare (Suttle and Chan, 1994). The abundances reported differed depending on the host used for the assay (Waterbury and Valois, 1993; Suttle and Chan, 1994; Marston and Sallee, 2003; Sullivan et al., 2003; Millard and Mann, 2006; Dekel-Bird et al., 2015). Thus, it is well known that these measures underestimate the number of infective cyanophages (Suttle, 2005; Baran et al., 2018) since it is impossible to use all possible host types in an assay.

A qPCR assay using primers with a low degree of degeneracy (Fuller et al., 1998) has been used to quantify a subset of the total T4-like cyanophages (Matteson et al., 2013). Abundances in the Sargasso Sea ranged from 3 × 10^3^ to 5 × 10^4^ ⋅ ml^–1^, and reached values as high as 4 × 10^5^ ⋅ ml^–1^ during a *Synechococcus* spring bloom in the South Pacific Ocean (Matteson et al., 2013). Measurements using the polony method, which quantified T4-like cyanophages at the family level in the NPSG, were considerably higher than these previous reports, generally by 1–2 orders of magnitude (Figures 6A,B). It should be noted that the polony method is better than other PCR-based methods (qPCR and droplet digital PCR) when quantification is done with moderate to high primer and probe degeneracy required to encompass the diversity of phages found at the family level (Baran et al., 2018).

Increases in cyanophage abundances are consistently found with increases in cyanobacterial numbers. This is irrespective of whether the entire population is enumerated using the polony method or different subsets of cyanophages are quantified using infective titer or qPCR assays. This is the case whether seasonal surveys, transects or depth profiles were analyzed (Waterbury and Valois, 1993; Suttle and Chan, 1994; Marston and Sallee, 2003; Matteson et al., 2013; Baran et al., 2018) and holds for the summer depth distribution of cyanophages in the NPSG (this study). However, the fairly even distribution of cyanophages over the water column in spring deviates from this generality. Even though a clear structure in depth distribution of *Prochlorococcus* was observed, both T4-like and T7-like cyanophage depth distribution was less structured. We consider this likely to be a result of recent deep mixing and propose that changes in cyanophage standing stocks may occur somewhat more slowly, lagging behind changes in cyanobacterial populations after such events.

The impact of viruses on aquatic ecosystems is often inferred from the concentration of viruses relative to microbial cells (Wigington et al., 2016). To advance our understanding of the influence of viruses on particular host taxa or functional groups it is imperative that we be able to quantify the viruses infecting these host types at the population level. Development of the polony method for the direct quantification of the two major virus families that infect the cyanobacteria (Baran et al., 2018, this study) is a major step toward this goal and has provided the first information on the absolute abundances of the two major cyanophages families with depth. Our first observations using the method raise interesting questions regarding the processes leading to their abundances at different depths in the oligotrophic NPSG. The differences in depth distribution patterns for cyanophages versus cyanobacteria, relative to VLPs versus bacteria, further highlight the importance of investigating components of the virus pool individually.

The polony method, developed for the quantification of T4-like and T7-like cyanophages (this study, Baran et al., 2018), is amenable to rapid collection of small volume samples that can be frozen and stored for many months prior to analyses. This will allow for surveying the abundances of these two cyanophage families over high temporal and spatial scales both in the North Pacific Ocean as well as in other oceanic regions. Furthermore, this method can be adapted for the quantification of other virus families, that infect different components of the Bacteria, Archaea, or Eukarya (see discussion in Baran et al., 2018). Importantly, it can be further adapted for high throughput determination of the extent to which these cyanophages, and indeed other viruses, infect their hosts in the environment through the interrogation of single cells for virus nucleic acids. As such, the application of the polony method at multiple scales will bring us closer to a quantitative understanding of the distribution patterns of different components of the virus community and the impacts on their hosts at a global scale.

## Data Availability Statement

The polony sequences reported here have been submitted to NCBI under the accession numbers MN701566–MN701576.

## Author Contributions

SG, NB, and DL developed the polony method for the T4-like cyanophages. YH and DL designed and sampled the field research. SG and YH carried out the experiments and analyzed the data. SG and DL wrote the manuscript.

## Conflict of Interest

The authors declare that the research was conducted in the absence of any commercial or financial relationships that could be construed as a potential conflict of interest.
