# Supplementary material for: Quantification of T4-Like and T7-Like Cyanophages Using the Polony Method Show They Are Significant Members of the Virioplankton in the North Pacific Subtropical Gyre
Source: Front Microbiol. 2020 Jun 16;11:1210. doi: 10.3389/fmicb.2020.01210 (PMC7308941; doi:10.3389/fmicb.2020.01210)
Supplement: Supplementary file 2 [file Table_1.pdf]

Supplementary Table 1: T4-like phages and environmental sequences used in phylogenetic analysis and primer design, and representatives of those used for probe design, with their *g20* nucleotide NCBI accession numbers.

	g20 sequence	Accession number	Usage
**T4-like cyanophages**	P-SSM4 *	AY940168	g20 primers and probes design
	Syn19 *	GU071106	
	S-SM1 *	GU071094	
	S-SSM5	GU071097	
	P-RSM4 *	GU071099	
	Syn33 *	GU071108	
	P-SSM7	GU071103	
	Syn9 *	DQ149023	
	S-ShM2	GU071096	
	P-TIM40 *	KP211958	
	S-PM2 *	AF363675	
	Syn1	GU071105	
	S-RSM4 *	FM207411	
	P-SSM2	AY939844	
	S-SM2 *	GU071095	
	S-SSM7 *	GU071098	
	P-HM1 *	GU071101	
	P-HM2 *	GU075905	
	P79	AY027983	
	S-WHM1	AF016385	
	S-LKM3 *	MN701563	
	P-TIM68 *	KM359505	
	S-BnM1	AF016386	
	S-CAM4 isolate 0809SB33	KU686201	
	S-RIM50	KU594605	
	S-RIM8 isolate RW_06_0613	KX349287	
	S-RIM32 isolate RW_108_0702	KU594606	
	S-CAM22 isolate 1209TA19	KU686209	
	S-MbCM7	KF156338	
	S-IOM18	HQ317383	
	S-MbCM100	KF156340	
	S-TIM4 *	MH512890	
	S-WAM2 isolate 0810PA29	KU686211	
	S-WAM1 isolate 0810PA09	KU686210	
	S-RIM44 isolate W2_07_0710	KU594607	
	S-SKS1	HQ633071	
	Syn2 *	EU715809	g20 probes design
	P-RSM1 *	HQ634175	
	S-SSM2 *	AGH57437	
	S-SSM1 *	EU715790	

* sequences used in phylogenetic analysis shown in Figure 1

	g20 sequence	Accession number	Usage
**T4-like cyanophages**	P-TIM3 *	JF837215	g20 probes design
	P-SSM9 *	EU715799	
	P-SSM12 *	EU715802	
	P-SSM11 *	EU715801	
	S-RIM6 *	AY259249	
	P-SSM10 *	EU715800	
	S-RIM10 *	AY259253	
	S-RIM7 *	AY259250	
	Syn30 *	HQ634189	
	P-TIM75 *	MN701564	
	P-RSM5 *	EU715798	
	P-SSM3 *	EU715786	
	P-SSM1 *	EU715797	
**Non-cyano T4-like phages**	T4 *	AF158101	
	HTVC008M *	KC465899	
	vB_EcoM_VR7 *	HM563683	
	RB32 *	DQ904452	
	RB69 *	AY303349	
	133 *	HM114315	
	Ac42 *	HM032710	
	31 *	AY962392	
	RB43 *	AY967407	
	KP15 *	GU295964	
	RB49 *	AY343333	
	Aeh1 *	AY266303	
	65 *	GU459069	
	nt-1 *	HQ317393	
	KVP40 *	AY283928	
	phiM12 *	KF381361	
**Non-cyano T4-like environmental sequences**	SS4055 *	AY028031	
	GS2624 *	AY027945	
	SS4850 *	AAK31775	
	GS2711 *	AY027956	
	SS4020 *	AY028018	
	SS4804 *	AY028065	
	GS2704 *	AY027952	
	SE36 *	AY028010	
	SE2 *	AY027986	
	24 *	MN701577	
	27 *	MN701578	
	23 *	MN701579	
	21 *	MN701580	
	26 *	MN701581	
	32 *	MN701582	
	28 *	MN701583	
	8 *	MN701584	
	uvDeep-GF2-KM20-C144 *	KT997822	

* sequences used in phylogenetic analysis shown in Figure 1

	g20 sequence	Accession number	Usage
**Polonies**	P38269 *	MN701566	Verification of the identity of polonies from this study
	P1811 *	MN701567	
	P149 *	MN701568	
	P41126 *	MN701569	
	P23153 *	MN701570	
	P92 *	MN701571	
	P2815 *	MN701572	
	P1520 *	MN701573	
	P24235 *	MN701574	
	P36266 *	MN701575	
	P2018 *	MN701576	

* sequences used in phylogenetic analysis shown in Figure 1
