# Supplementary material for: Quantification of T4-Like and T7-Like Cyanophages Using the Polony Method Show They Are Significant Members of the Virioplankton in the North Pacific Subtropical Gyre
Source: Front Microbiol. 2020 Jun 16;11:1210. doi: 10.3389/fmicb.2020.01210 (PMC7308941; doi:10.3389/fmicb.2020.01210)
Supplement: Supplementary file 1 [file Data_Sheet_1.PDF]

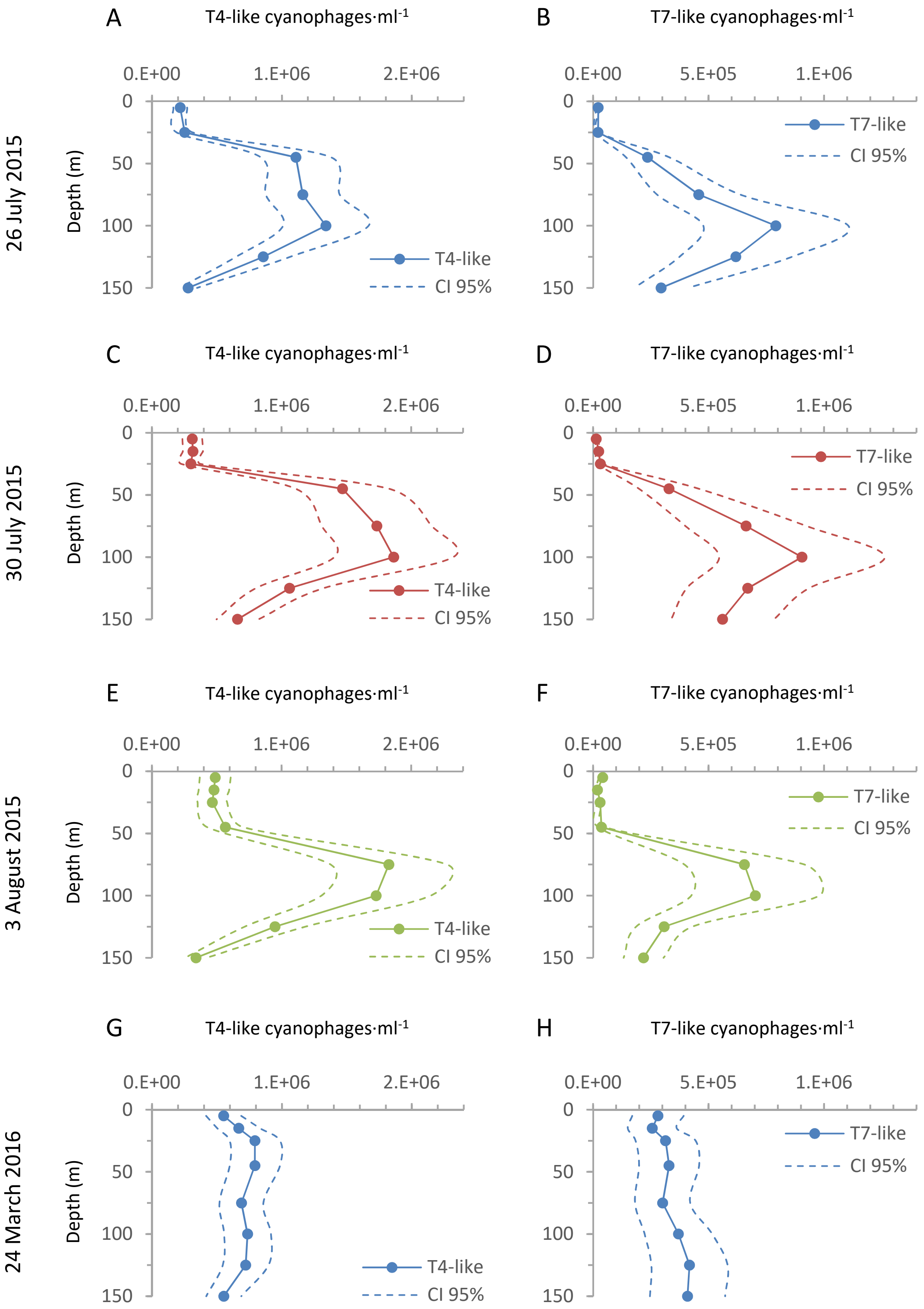

Supplementary Figure 1: Depth distribution of cyanophages in the NPSG with 95% confidence intervals. Abundances of T4-like cyanophages (left panels) and T7-like cyanophages (right panels) during the summer cruise on 26 July 2015 (A, B), 30 July 2015 (C, D), 3 August 2015 (E, F) and during the spring cruise on 24 March 2016 (G-H). Symbols and solid lines indicate cyanophage abundances determined by the polony method. Dashed lines indicate 95% confidence intervals.
